# The timing of ostomy closure in infants with necrotizing enterocolitis: a systematic review

**DOI:** 10.1007/s00383-012-3091-9

**Published:** 2012-04-21

**Authors:** Marie-Chantal Struijs, Cornelius E. J. Sloots, Wim C. J. Hop, Dick Tibboel, Rene M. H. Wijnen

**Affiliations:** 1 Department of Pediatric Surgery, Erasmus MC-Sophia, PO Box 2060, 3000 CB Rotterdam, The Netherlands; 2 Department of Biostatististics, Erasmus University Rotterdam, Rotterdam, The Netherlands

**Keywords:** Ostomy closure, Complications, Infants, Necrotizing enterocolitis, Systematic review

## Abstract

**Purpose:**

The optimal timing of ostomy closure is a matter of debate. We performed a systematic review of outcomes of early ostomy closure (EC, within 8 weeks) and late ostomy closure (LC, after 8 weeks) in infants with necrotizing enterocolitis.

**Methods:**

PubMed, EMbase, Web-of-Science, and Cinahl were searched for studies that detailed time to ostomy closure, and time to full enteral nutrition (FEN) or complications after ostomy closure. Patients with Hirschsprung’s disease or anorectal malformations were excluded. Analysis was performed using SPSS 17 and RevMan 5.

**Results:**

Of 778 retrieved articles, 5 met the inclusion criteria. The median score for study quality was 9 [range 8–14 on a scale of 0 to 32 points (Downs and Black, J Epidemiol Community Health 52:377–384, 1998)]. One study described mean time to FEN: 19.1 days after EC (*n* = 13) versus 7.2 days after LC (*n* = 24; *P* = 0.027). Four studies reported complication rates after ostomy closure, complications occurred in 27 % of the EC group versus 23 % of the LC group. The combined odds ratio (LC vs. EC) was 1.1 [95 % CI 0.5, 2.5].

**Conclusion:**

Evidence that supports early or late closure is scarce and the published articles are of poor quality. There is no significant difference between EC versus LC in the complication rate. This systematic review supports neither early nor late ostomy closure.

## Introduction

Ostomy formation is inevitable in certain cases, for example in almost half the patients operated on for necrotizing enterocolitis (NEC) [1]. Unfortunately, in 15–68 % of cases ostomy-related complications may occur, such as stricture, parastomal hernia, prolapse, wound infection, wound fistula, wound dehiscence, and small bowel obstruction [2–5]. Especially premature infants are at a high risk; in patients with necrotizing enterocolitis, lower gestational age and birth weight were associated with greater risk of ostomy related complications [3]. Subsequent ostomy closure carries a complication rate of about 20 %, including wound infection, wound dehiscence, enterocutaneous fistula, bowel obstruction, anastomotic leak, and anastomotic stricture [2, 5, 6].

Following ostomy formation, surgeons tend to delay ostomy closure for at least 8 weeks or until the infant weighs 2 kg because of surgical aspects such as the postoperative abdominal adhesions and anesthetic aspects such as morbidity associated with ventilation anticipated in case of earlier closure [7–9]. The timing of ostomy closure is highly variable based on the surgeon’s preference or local protocols, however, universally without evidence based practice. Early closure could not only avoid ostomy-related complications but it could also be favorable since having an ostomy is associated with diarrhea, severe fluid and electrolyte losses, and growth retardation [10]. Moreover, ostomy closure during the same hospital admission is also favorable for parents and caregivers.

Therefore, we performed a systematic review to find an answer to the question whether early or late ostomy closure is preferred in infants with a history of NEC. The outcome measures were, time to full enteral nutrition and the complication rate.

## Methods

### Search strategy

We conducted a systematic literature search in the PubMed, EMbase, Web-of-Science, and Cinahl databases from 1966 to October 2010. The following search terms were applied for the PubMed database: (stoma[tw] OR stomata[tw] OR stomas[tw] OR stomy[tw] OR ostom*[tw] OR enterostom*[tw] OR cecostom*[tw] OR coecostom*[tw] OR caecostom*[tw] OR colostom*[tw] OR duodenostom*[tw] OR ileostom*[tw] OR jejunostom*[tw]) AND (clos*[tw] OR seal*[tw] OR restor*[tw] OR repair*[tw] OR recover*[tw] OR re-establ*[tw] OR cure*[tw]) AND (infan*[tw] OR newborn*[tw] OR neonat*[tw]). The other databases were searched with the appropriate search terms concerning ostomy closure in infants less than 2 years of age. No limits were applied. All retrieved article titles and subsequent abstracts were screened for eligibility by two independent reviewers (MCS and CEJS). Bibliographies of all selected abstracts were screened to identify any additional trials.

### Selection criteria

All studies that compared early versus late ostomy closure in infants were eligible for inclusion in this study. In addition, at least two-thirds of included patients should be diagnosed with necrotizing enterocolitis and included studies needed to contain a description of either complication rate after ostomy closure and/or time to full enteral nutrition (FEN) after ostomy closure. Studies involving patients with Hirschsprung’s disease or anorectal malformations were excluded, because the timing of ostomy closure is not related to the patient’s recovery but to the moment of institutional-determined surgical repair of either the Hirschsprung’s disease or anorectal malformation.

Early ostomy closure (EC) was defined as ostomy closure within 8 weeks after ostomy formation; late ostomy closure (LC) as ostomy closure more than 8 weeks later than the ostomy formation. Reason being that in our hospital the 8 weeks time point is considered the cutoff point, without formal evidence from the literature.

### Quality assessment

Study quality was assessed with a checklist as proposed by Downs et al. [11]. This checklist contains 27 questions in 5 domains: reporting, external validity, internal validity-bias, internal validity-confounding, and power. Optimal study quality scores were 32 points.

### Data extraction

Two reviewers (MCS, CEJS), blinded for each other’s results, extracted the following predefined data: study design, study population, time to ostomy closure, complications following ostomy closure (including wound infection, fistula, wound dehiscence, wound evisceration, bowel obstruction, and anastomotic obstruction), and time to full enteral nutrition after ostomy closure. Discrepancies were resolved by consensus after discussion.

### Statistical analysis

Data were analyzed using SPPS (version 17; SPSS, Chicago, IL) and Review Manager (RevMan) software version 5.0 (Copenhagen: The Nordic Cochrane Center, The Cochrane Collaboration, 2008) was used to pool data from the studies for the meta-analysis. Comparisons of dichotomous data were carried out using the Mantel–Haenszel statistical method under assumption of fixed effect analysis model, which was derived from the fact that included studies entail similar therapies. Results for comparisons of dichotomous outcomes were expressed as odds ratio (OR) with 95 % confidence interval (CI). Heterogeneity of the data was tested using a χ^2^ statistic. All statistical tests were performed at 5 % significance level.

## Results

### Trial flow for manuscript selection

**Fig. 1:**
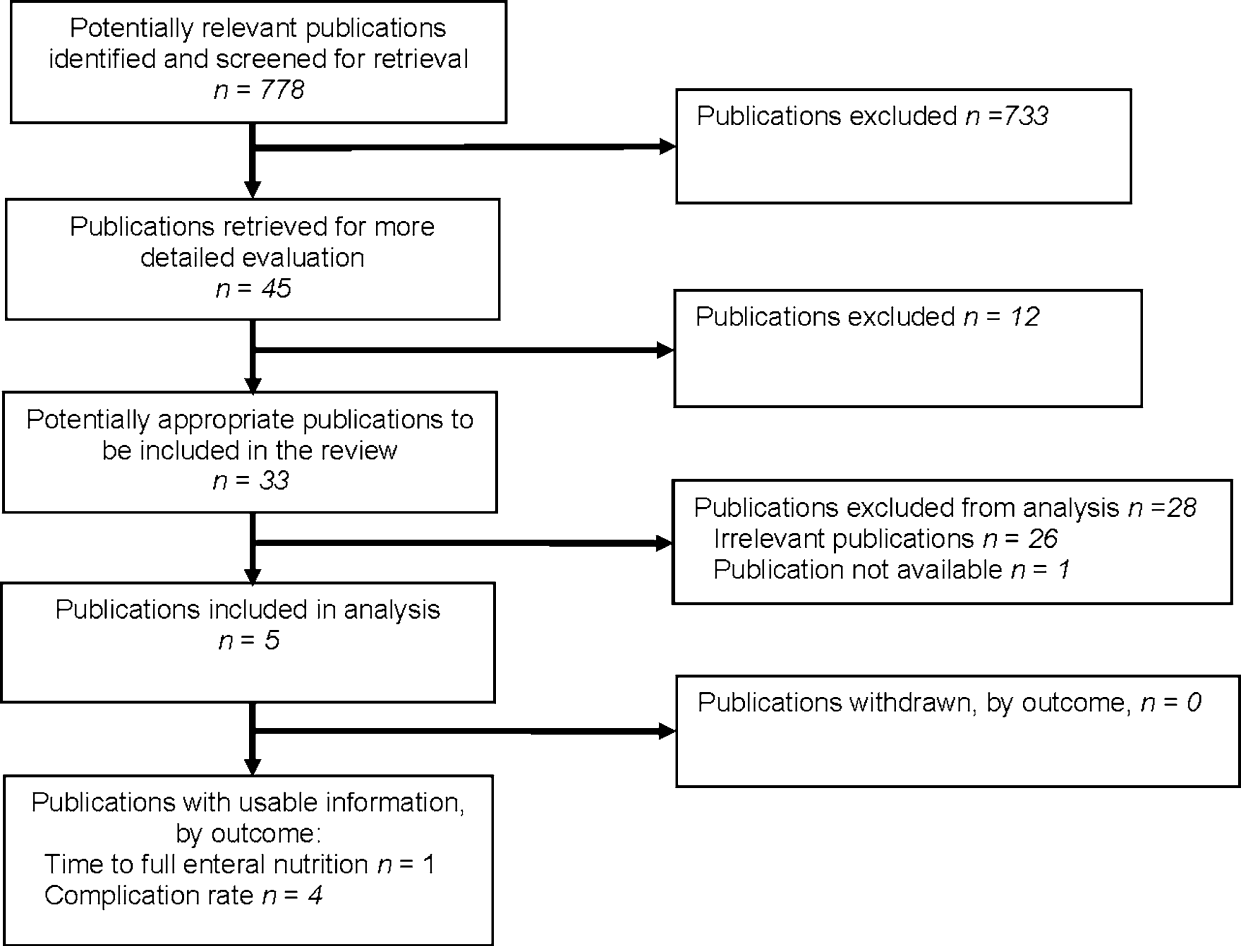
PRISMA flow chart presenting the selection of studies

The searches yielded 778 articles, of which 733 were found irrelevant based on the title. Of the 45 remaining articles, 33 were potentially eligible for inclusion in the meta-analysis (Fig. 1). Of these, five articles met the selection criteria and were included in this study. Four studies compared complication rates after ostomy closure and only one study focused on mean time to full enteral nutrition after ostomy closure.

### Characteristics of included studies

The characteristics of the five included studies are described in Table 1. Except for the study of Gertler et al. [14] which was a prospective cohort study, all studies described retrospective cohorts. Sample size ranged from 10 to 92 patients and the five studies concerned in total 253 patients, 160 of whom underwent EC and 93 underwent LC. The gestational age of the patients varied between 25 and 41 weeks. The main type of ostomy was ileostomy (between 54 and 100 %). Evaluation of the distal segment for strictures was done in four of the five studies, either preoperative with contrast rontgenography or during ostomy closure. The poorest study scored 8 points for study quality; the best study 14 points (median 9 points).

**Table 1:** Included publications

Author	Journal of Publication	Year	Study design	*n*		% Diagnosis NEC	Type of ostomy *n* (%)			Study quality^c^
				EC	LC		J	I	C	
Al-Hudhaif [12]	J Pediatr Surg	2009	Retrospective cohort study	13	24	100	4 (11)	28 (76)	5 (13)	13
Weber [13]	Arch Surg	1995	Retrospective cohort study	92	–	72^a^	29 (32)	50 (54)	13 (14)	9
Gertler [14]	J Pediatr Surg	1987	Prospective cohort study	3	7	100		10 (100)		8
Musemeche [15]	J Pediatr Surg	1987	Retrospective cohort study	39	50	100	10 (10)^b^	75 (75)^b^	15 (15)^b^	14
Cogbill [16]	Surg Gynecol Obstet	1985	Retrospective cohort study	13	12	100	3 (12)	16 (64)	6 (24)	9

*EC* indicates early ostomy closure, *LC* late ostomy closure, *J* jejunostomy, *I* ileostomy, *C* colostomy

^a^This number is an indication, 72 % of 109 patients with ostomy had NEC, 17 infants died before ostomy closure. Separate number for total number of patients with NEC at ostomy closure were not provided

^b^In total 100 patients were included but time to ostomy closure was only provided for 89 patients, unfortunately no data were provided to separate these in type of ostomy

^c^Study quality as measured by the checklist in Downs et al. (optimal study quality scores were 32 points)

### Time to full enteral nutrition

The mean time to full enteral nutrition (FEN) was reported in one study. In the study by Al-Hudhaif et al. [12] FEN was 19.1 days (*n* = 13) in the EC group versus 7.2 days (*n* = 24) in the LC group (*P* = 0.027).

### Complications

**Fig. 2:**
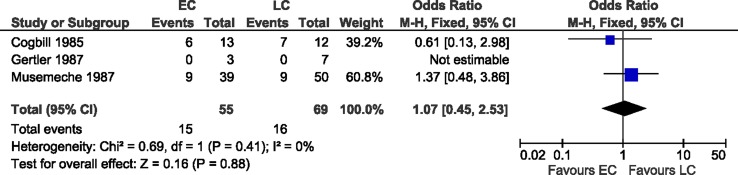
Forest plot comparison of postoperative complications in EC versus LC

Weber et al. [13] only analyzed EC, and found a complication rate of 39 % (Table 2); therefore, this study could not be used in the meta-analysis. The other three studies could be used for meta-analysis in the forest plot (Fig. 2). Combining all three studies, the complication rate did not differ greatly between both groups, 27 % (15/55) in the EC group versus 23 % (16/69) in the LC group. The combined odds ratio (LC vs. EC) was 1.1 [95 % CI 0.5, 2.5].

**Table 2:** Complication rate in early and late ostomy closure group

Study	Early ostomy closure			Late ostomy closure		
	*n*	Mean time to closure (days)	Complications *n* (%)	*n*	Mean time to closure (days)	Complications *n* (%)
Weber [13]	92	40	36 (39)	–	–	–
Gertler [14]	3	37	0 (0)	7	131	0 (0)
Musemeche [15]	39	31	9 (23)	50	112	9 (18)
Cogbill [16]	13	56	6 (46)	12	154	7 (58)

## Discussion

This systematic review showed that complication rate did not differ between early and late closure of ostomy in patients with necrotising enterocolitis. Only one study provided data on enteral feeding after ostomy reversal favoring late closure. Al-Hudhaif et al. [12] found a longer duration to achieve full enteral nutrition in the EC group (19.1 days in the EC group vs. 7.2 days in the LC group). These results were not comparable with another study, which found that when ostomy closure occurred at a mean time of 30 days, the mean time to full enteral nutrition was 8 days [17]. This study was excluded from the analysis because only 37 % of the included infants were diagnosed with necrotizing enterocolitis. Unfortunately, due to the limited number and relatively low quality of the studies, a systematical analysis of the mean time to full enteral nutrition after ostomy closure was not possible. This systematic review did not bring conclusive evidence on the most favorable timing of ostomy closure in infants with a history of necrotising enterocolitis.

Early closure is also associated with several other advantages. For one, maintaining a normal fluid and electrolyte balance is best helped by restoration of enterocolonic continuity as soon as possible. This was illustrated by six cases, as reported by Rothstein et al. [10], in which an ileostomy for NEC was complicated by chronic diarrhea, feeding difficulties, sepsis, rickets and developmental delay. These infants were all readmitted within the next 3 months due to severe acidosis and dehydration associated with a large-volume ileostomy output. This was resolved after reanastomosis, which illustrates the potential benefit of early ostomy closure. Another advantage of early ostomy closure was the possible prevention of distal strictures. The observed rate of distal strictures after ostomy formation was around 40 % [2]. Early closure of the ostomy might lead to fewer strictures caused by feedings. This is speculative, however, and should be proven by a randomized controlled trial.

The results of our review should be interpreted with caution given the small sample sizes of individual studies and given the fact that not all studies included an early and late ostomy closure group. Also, the quality of the studies is generally low, mainly due to the mostly retrospective nature of the studies. The data for the meta-analysis regarding complications came from three studies only and full-fledged analysis for the time to full enteral nutrition was not even possible. It would also be interesting to construct a receiver-operator characteristic to obtain the most favorable timing of ostomy closure. Unfortunately this was not possible due to limited availability of data points. It is also clear that the type of ostomy has significant impact on the outcome and need for undoing. Since different types of ostomies were included in the studies, the interpretation of the data is more difficult. A jejunostomy is usually associated with an extremely high output with electrolyte disturbances and poor absorption of nutrients and need for early undiversion. A well-managed distal ileostomy or colostomy is usually well tolerated with few metabolic consequences and no urgent need for ostomy closure. If the presented patient series were broken-up in different ostomy categories, the numbers would have been too small to make any conclusions.

A randomized controlled trial (RCT) could bring conclusive evidence comparing early versus late ostomy closure in terms of time to full enteral feeding, weight gain, complication rate, and duration of hospital stay. Patients should be stratified according to ostomy type. Unfortunately, no RCTs were available, and this is a problem encountered very often in pediatric surgery [18]. Comparing laparotomy versus laparoscopy for pyloric stenosis has been the subject of many studies, a meta-analysis could even be performed for this topic [19–21]. Same counts for different kinds of fundoplication in gastro-esophageal reflux disease [22, 23]. In infants with necrotizing enterocolitis, the main focus of the studies was peritoneal drain versus laparotomy [24–26]. Unfortunately, there are no RCTs available regarding optimal timing of ostomy closure. Since no RCT is available in infants, we reviewed the data of adults regarding timing of ostomy closure. For comparison, in adults with temporary ostomy due to trauma or colorectal surgery, it is considered safe to reverse ostomy on a short time notice. Therefore, this could endorse the safety of earlier closure in infants too.

In conclusion, early closure (<8 weeks) of an ostomy in infants did not lead to more surgery-related complications. A recommendation for early or late ostomy closure cannot be given on the basis of the data from five studies of low quality. Other factors such as parent burden should also play a role in the strategy of timing of ostomy closure.
